# A transplantable tumor model allowing investigation of NY-BR-1-specific T cell responses in HLA-DRB1*0401 transgenic mice

**DOI:** 10.1186/s12885-019-6102-6

**Published:** 2019-09-13

**Authors:** Krishna Das, David Eisel, Mathias Vormehr, Karin Müller-Decker, Adriane Hommertgen, Dirk Jäger, Inka Zörnig, Markus Feuerer, Annette Kopp-Schneider, Wolfram Osen, Stefan B. Eichmüller

**Affiliations:** 1 Research Group GMP & T Cell Therapy, German Cancer Research Center (DKFZ), Heidelberg, Germany; 2 Division of Virology, Innsbruck Medical University, Innsbruck, Austria; 3 Faculty of Biosciences, University Heidelberg, Heidelberg, Germany; 4 Biopharmaceutical New Technologies (BioNTech) Corporation, Mainz, Germany; 5 University Medical Center of the Johannes Gutenberg University, Mainz, Germany; 6 Core Facility Tumor Models, German Cancer Research Center (DKFZ), Heidelberg, Germany; 7 Department of Molecular & Radiation Oncology, German Cancer Research Center (DKFZ), Heidelberg, Germany; 8 CCU Applied Tumor Immunity, German Cancer Research Center (DKFZ), Heidelberg, Germany; 9 Department of Medical Oncology, National Center for Tumor Diseases (NCT) and University Hospital Heidelberg, Heidelberg, Germany; 10 Institute of Immunology, Regensburg Center for Interventional Immunology (RCI), University Regensburg and University Hospital Regensburg, Regensburg, Germany; 11 Department of Biostatistics, German Cancer Research Center (DKFZ), Heidelberg, Germany

**Keywords:** NY-BR-1, Differentiation antigen, CTL epitope, HLA transgenic mice

## Abstract

**Background:**

NY-BR-1 has been described as a breast cancer associated differentiation antigen with intrinsic immunogenicity giving rise to endogenous T and B cell responses. The current study presents the first murine tumor model allowing functional investigation of NY-BR-1-specific immune responses in vivo.

**Methods:**

A NY-BR-1 expressing tumor model was established in DR4tg mice based on heterotopic transplantation of stable transfectant clones derived from the murine H2 compatible breast cancer cell line EO771. Composition and phenotype of tumor infiltrating immune cells were analyzed by qPCR and FACS. MHC I binding affinity of candidate CTL epitopes predicted in silico was determined by FACS using the mutant cell line RMA-S. Frequencies of NY-BR-1 specific CTLs among splenocytes of immunized mice were quantified by FACS with an epitope loaded D^b^-dextramer. Functional CTL activity was determined by IFNγ catch or IFNγ ELISpot assays and statistical analysis was done applying the Mann Whitney test. Tumor protection experiments were performed by immunization of DR4tg mice with replication deficient recombinant adenovirus followed by s.c. challenge with NY-BR-1 expressing breast cancer cells.

**Results:**

Our results show spontaneous accumulation of CD8^+^ T cells and F4/80^+^ myeloid cells preferentially in NY-BR-1 expressing tumors. Upon NY-BR-1-specific immunization experiments combined with in silico prediction and in vitro binding assays, the first NY-BR-1-specific H2-D^b^-restricted T cell epitope could be identified. Consequently, flow cytometric analysis with fluorochrome conjugated multimers showed enhanced frequencies of CD8^+^ T cells specific for the newly identified epitope in spleens of immunized mice. Moreover, immunization with Ad.NY-BR-1 resulted in partial protection against outgrowth of NY-BR-1 expressing tumors and promoted intratumoral accumulation of macrophages.

**Conclusion:**

This study introduces the first H2-D^b^-resctricted CD8^+^ T cell epitope-specific for the human breast cancer associated tumor antigen NY-BR-1. Our novel, partially humanized tumor model enables investigation of the interplay between HLA-DR4-restricted T cell responses and CTLs within their joint attack of NY-BR-1 expressing tumors.

**Electronic supplementary material:**

The online version of this article (10.1186/s12885-019-6102-6) contains supplementary material, which is available to authorized users.

## Background

Although improved diagnostic tools and advanced therapy approaches have succeeded in reducing the standardized cancer mortality rate of breast cancer over the last decades, this tumor entity still remains the second most frequent cancer type among women, predicted to cause more than 93,000 deaths in the European Union in the year 2020 [1]. New treatment strategies are thus needed in order to further improve the clinical outcome of breast cancer patients, particularly since standard therapy approaches are often associated with severe side effects [2] or can even induce therapeutic resistance [3]. At this point, immunological treatment strategies exploiting the activity of autologous tumor-reactive T cells might offer an attractive complement or alternative to classical treatment regimens. In fact, adoptive T cell therapy, particularly in combination with checkpoint inhibitors, has yielded promising results in various clinical settings [4, 5]. The concept of this therapeutic approach relies on the notion that activated tumor antigen-specific T cells selectively attack tumor cells leaving healthy tissue unaffected. However, to fulfill this aim, target antigens need to be identified that are selectively expressed by the tumor, but absent or underrepresented in normal tissue. The breast cancer associated differentiation antigen NY-BR-1 meets this criterion as it was found to be strongly overexpressed in breast tumors originating from ductal epithelial cells [6].

Interestingly, NY-BR-1 is characterized by marked intrinsic immunogenicity as both antibody [7] and cytotoxic T cell (CTL) responses [8, 9] have been detected in breast cancer patients. CTLs recognize short peptides that are presented by MHC I molecules on the surface of the target cell, resulting in apoptosis induction within the recognized cell [10, 11]. Thus, most T cell based immunotherapy approaches have aimed at the induction of tumor-reactive CD8^+^ T cells, considered as ultimate effector cells due to their capacity of direct tumor cell killing. On the other hand, tumor antigen-specific CD4^+^ T cells have been demonstrated to represent essential contributors of CTL-mediated tumor attack [12], implying that epitopes presented by MHC II molecules to CD4^+^ T cells are of central importance in tumor immune surveillance [13]. In a previous study we thus used HLA-DR4 transgenic (HLA-DR4tg) mice to identify the first NY-BR-1-specific CD4^+^ T cell epitopes that might help to induce and monitor tumor antigen-specific T cell responses in breast cancer patients [14]. However, a NY-BR-1 expressing tumor model allowing investigation of NY-BR-1-specific T cell responses in vivo has been lacking so far. We thus set out to establish such a tumor model based on ectopic transplantation of a NY-BR-1 expressing mouse mammary carcinoma cell line onto HLA-DR4tg mice. Moreover, we introduce the first NY-BR-1-specific H2^b^-restricted CTL epitope and describe the composition of tumor infiltrating immune cell populations with particular focus on NY-BR-1-specific CD4^+^ T cells and HLA-DR4 positive tumor-associated macrophages. The NY-BR-1 expressing tumor model presented here, offers a valuable tool to investigate immune responses against NY-BR-1 expressing tumor cells in vivo.

## Methods

### Cell lines

Both murine tumor cell lines used were of C57BL/6 origin (H2^b^). The mammary adenocarcinoma cell line EO771 was purchased from TEBU-Bio (Offenbach, Germany) and RMA-S cells (a Rauscher virus induced T lymphoma) were kindly provided by Günter Hämmerling, DKFZ Heidelberg, Germany. RMA-S cells were propagated in complete RPMI medium containing RPMI 1640 supplemented with Glutamax (Life technologies / Thermo Fisher), 10% FCS (Biochrom, Berlin, Germany), and 1% penicillin-streptomycin (Life technologies / Thermo Fisher). EO771 cells were cultured in complete RPMI medium containing 1 mM HEPES buffer (Sigma-Aldrich, Saint Louis, MO).

### HLA-DR4-transgenic mice

B6.129S2-*H2-Ab1*^*tm1Gru*^ Tg (HLA-DRA/H2-Ea,HLA-DRB1*0401/H2-Eb)1Kito mice expressing a chimeric HLA-DRA-IE^d^α/HLA-DRB1*0401-IE^d^β molecule on a H2-IA^0/0^ background [15] (designated as HLA-DR4tg mice throughout this paper) were obtained from Taconic (Cologne, Germany) and further bred in the Centralized Laboratory Animal Facilities of the German Cancer Research Center Heidelberg. Animals were group housed in standard individually ventilated cages with wood chip embedding (LTE E-001, ABEDD, Vienna, Austria), nesting material, ad libitum diet (autoclaved mouse/rat housing diet 3437, PROVIMI KLIBA AG, Kaiseraugst, Switzerland) and autoclaved tap water.

In accordance with the Appendix A of des European Convention for the Protection of Vertebrate Animals used for Experimental and Other Scientific Purposes from 18th March 1986 room temperature and relative humidity were adjusted to 22.0 ± 2.0 °C and 55.0 ± 10.0%, respectively. All animals were housed under strict specified pathogen-free (SPF) conditions according to the recommendations of the FELASA. The light/dark (L/D) cycle was adjusted to 14 h lights on and 10 h lights off with the beginning of the light and dark period set at 6.00 am and 8.00 pm, respectively.

All animal experimentation performed in this study was conducted according to the national guidelines and was reviewed and confirmed by the institutional review board/ethics committee of the German Cancer Research Center, Heidelberg). The animal experiments were finally approved by the responsible national authority, which is the Regional Authority of Karlsruhe (Germany; official approval ID 35–9158.81/G172–12).

Sample size calculation was performed by the Biostatistics Department of the DKFZ following standard procedures. Mice were randomized to the different treatment groups. Treatment was performed in random order. Health status of mice has regularly been tested by the Animal Core Facility. Only animals with approved health status were included in the experiments.

### Generation of stable NY-BR-1 expressing transfectant clones

EO771 cells were transfected with 1.2 μg linearized pcDNA3.1(−)zeo-NY-BR-1 expression vector generated upon cloning of the NY-BR-1 encoding cDNA fragment from pcDNA3.1-NY-BR-1 (kindly provided by I. Zörnig) into pcDNA3.1(−)zeo (Invitrogen / ThermoFisher, Waltham, MA) via Kpn1/Not1 digestion. After selection with Zeocin (400 μg/mL), individual clones were raised by limiting dilution.

### Western blot analysis

Cellular proteins (15–50 μg) of heat denatured cell lysates were separated by SDS PAGE using a 10% polyacrylamide gel, followed by electro-transfer onto nitrocellulose membranes. Membranes were incubated overnight at 4 °C with a murine monoclonal antibody (clone#2, diluted 1:1000) specific for NY-BR-1 in 0.5% non-fat milk in Tris buffered saline containing 0.1% Tween 20 (TBS-T buffer) on a shaking platform. Beta actin was detected using a monoclonal antibody (MP Biomedical, Solon, OH) diluted 1:10,000 in 0.5% non-fat milk in TBS-T buffer. Next, membranes were washed and incubated with horseradish peroxidase- conjugated secondary antibody (Santa Cruz Biotechnology, Santa Cruz, TX) diluted 1:10,000 in 0.5% non-fat milk in TBS-T buffer for 1 h at room temperature. Protein signals were detected using the enhanced chemiluminescence system (GE Healthcare, Munich, Germany) either by exposing blots to an X-ray film or by a CCD camera.

### Peptide binding assay

Peptide binding assays were performed as described [16]. Briefly, 2 × 10^5^ RMA-S cells were incubated overnight with graded peptide concentrations in round bottom microtiter plates followed by indirect immunoflourescence staining using supernatants of hybridoma E3–25 or B22.249 specific for H2-K^b^ or H2-D^b^ molecules, respectively. All hybridomas were kindly provided by G. Hämmerling.

### IFNγ ELISPOT assay

IFNγ secretion of splenocytes from immunized mice or of established T cell lines was analyzed by IFNγ ELSIPOT assay as described previously [17], except that 5 μg/mL anti-mouse IFNγ capture antibody were used for membrane coating. ELISPOT results were analyzed using two ELISPOT reader devices from AID (Strassberg, Germany) or C.T.L. (Cleveland, OH), respectively. Statistical analysis was performed using Mann Whitney test.

### IFNγ catch assay

Flow cytometric detection of IFNγ secreting T cell subpopulations was performed using the Mouse IFN-γ Secretion Assay – Detection Kit (Miltenyi Biotec GmbH, Gladbach, Germany) according to the optimized manufacturer’s protocol. Briefly, 2 × 10^6^ - 2.5 × 10^6^ spleen cells were stimulated overnight with 5 μg/mL peptide. Next day, cells were washed twice followed by incubation with IFNγ catch reagent for 2–3 h at 37 °C cells. Cells were then washed and stained with LIVE/DEAD® Fixable Yellow Dead Cell Stain or LIVE/DEAD® Fixable Blue Dead Cell Stain Kit (Invitrogen / Thermo Fisher) diluted 1:1000 in PBS for 30 min at 4 °C. Finally, cells were stained with fluorochrome-labelled anti-mouse CD4 antibody, anti-mouse CD8 antibody and anti-IFN-γ PE (Miltenyi Biotec GmbH, Gladbach, Germany). Data was acquired on a FACS Calibur1, FACS Canto II or LSR II and analyzed with FlowJo software. Statistical analysis was done using Mann Whitney test. Finally, cells were stained with fluorochrome-labelled anti-mouse CD4 antibody, anti-mouse CD8 antibody and anti-IFN-γ PE (Miltenyi Biotec GmbH, Gladbach, Germany). Data was acquired on a FACS Calibur1, FACS Canto II or LSR II and analyzed with FlowJo software. Statistical analysis was done using Mann Whitney test.

### Tumor growth experiments

Harvested tumor cell lines were washed three times in PBS and adjusted to titers mentioned in the Results part. Tumor cells were suspended in PBS (100 μL) and injected subcutaneously into the right hind flank of 6–10 week-old female HLA-DR4tg mice without anaesthesia. Tumor growth was monitored by caliper measurements twice per week. Mice were killed by CO2 intoxication 30 days after tumor cell injection or when tumors reached a size of 15 mm in diameter, respectively.

### Immunization with recombinant adenovirus

Recombinant, replication-deficient adenovirus encoding NY-BR-1 (Ad.NY-BR-1) and the empty control virus (Ad.Control) were purchased from GeneCust (Dudelange, Luxembourg). Mice were injected (i.p.) with 5 × 10^8^ plaque-forming units (pfu) Ad.NY-BR-1 or Ad. Control, respectively, and killed 14 days later by CO2 intoxication for splenectomy, unless otherwise indicated.

### Isolation of tumor infiltrating leukocytes

Excised tumors were cut into small pieces followed by digestion with a mixture of Collagenase D (0.5 mg/mL) (Roche Diagnostics, Mannheim, Germany), DNAse I (10 μg/mL) (Sigma-Aldrich), TLCK inhibitor (0.1 μg/mL) (Sigma-Aldrich, Saint Louis, U.S) and HEPES buffer (10 mM) (Sigma-Aldrich) in HBSS (Sigma-Aldrich) for 1 h at 37 °C. The tumor pieces were then passed through a 70 μm cell strainer and the resulting suspension was centrifuged at 1400 rpm for 10 min. Cells were resuspended in 5 mL RPMI medium and separated by density gradient centrifugation (Lympholyte M, Cedarlane Labs, Burlington, Canada). Leukocytes were harvested from the interphase and used for subsequent experiments. The pellet consisting of tumor cells was washed with PBS and used for RNA and protein isolation as required.

### Magnetic activated cell sorting (MACS) for positive selection

Cytotoxic T cells and tumor-infiltrating myeloid cells were enriched by magnetic cell sorting using magnetic antibody-coated (CD8a- or CD11b-specific) microbeads and MS or LS columns, respectively, according to the manufacturer’s instructions (Miltenyi Biotec).

### Dextramer staining

Spleen cell suspensions prepared from immunized mice were adjusted to 1 × 10^6^ cells/mL and incubated with LIVE/DEAD® Fixable Yellow Dead Cell Stain (Life Technologies / Thermo Fisher) diluted 1:1000 in PBS for 30 min at 4 °C. After washing with PBS, cells were incubated with APC-labeled H2-D^b^ dextramers (Immudex, Copenhagen, Denmark) loaded with NY-BR-1_1241-1249_ or with Human Papilloma Virus (HPV) 16 E7_49–57_, diluted as indicated in 60 μL FACS buffer for 30 min at room temperature in the dark. Fluorochrome-labeled antibodies against CD3 (clone 17A2), CD8 (clone 53–6.7), CD4 (clone GK1.5) CD14 (clone Sa14–2) (all purchased from Biolegend, San Diego, CA), and the respective isotype controls were diluted 1:50 in 60 μL FACS buffer and added to the cells without washing off the dextramers so that the final dilution of the antibodies was 100-fold. Cells were incubated with the antibody mix at 4 °C for 30 min. The cells were then washed and analyzed by flow cytometry for viable CD3^+^CD14^−^CD8^+^ dextramer^+^ cells.

### Flow cytometry and cell sorting

For flow cytometry, 1 × 10^6^ cells were used per sample unless otherwise indicated. A mix of 0.05 mg/mL purified rat anti-mouse CD16/CD32 (BD Pharmingen), rat serum (GeneTex, Irvine, CA) and hamster serum (Jackson Immunoresearch, West Grove, PA) was used to block Fc receptors on tumor infiltrating leukocytes for 20 min at 4 °C. This step was omitted for tumor cell lines and splenocytes. The cells were then washed twice and 100 μL of LIVE/DEAD® Fixable Yellow Dead Cell Stain or LIVE/DEAD® Fixable Blue Dead Cell Stain Kit, for UV excitation (Life technologies / Thermo Fisher) diluted 1:1000 in PBS was added. After incubation for 30 min at 4 °C, washed cells were stained with antibodies (or isotype controls) listed in Additional file 1: Table S1. After incubation for 1 h at 4 °C, cells were washed and resuspended in 200–400 μL FACS buffer for flow cytometric sorting or analysis, respectively. For intracellular cytokine staining, cells were incubated in 100 μL fixation permeabilization solution (BD Biosciences, San Jose, CA) for 20 min at 4 °C, followed by two washing steps using BD Perm/Wash buffer. Cells were then incubated for 1 h at 4 °C with 100 μL BD Perm/Wash buffer containing antibodies or isotype controls, respectively, diluted 1:100. Finally, cells were resuspended in 200–400 μL FACS buffer for analysis.

### Quantitative RT-PCR

Gene expression analysis by standard qRT-PCR using SYBR green was performed for the analysis of the polarization status of tumor-associated macrophages. Primer sequences are listed in Additional file 1: Table S2.

## Results

### Generation of a NY-BR-1 expressing breast cancer cell line

**Fig. 1:**
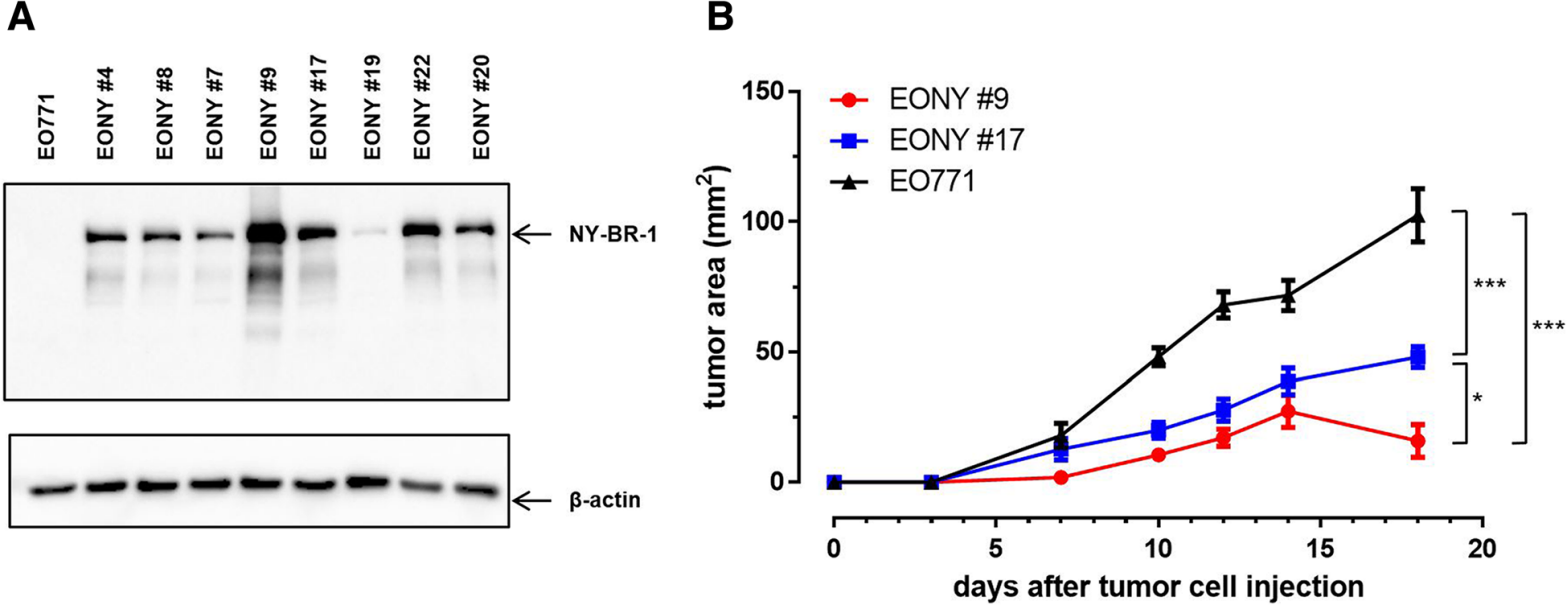
Characterization of EO771/NY-BY-1 transfectant clones. EO771/NY-BR-1 transfectant clones generated by transfection of EO771 cells with a linearized plasmid pcDNA3.1-NY-BR-1 followed by limiting dilution were characterized for NY-BR-1 expression in vitro and the ability to form tumors in vivo. **a** NY-BR-1 protein expression (159 kDa) in the selected clones was analyzed by Western blot. β-actin (42 kDa) was used as a loading control. **b** HLA-DRB1*0401tg mice were injected s.c. on the right flank with 2 × 10^5^ EO771, EONY #9 or EONY #17 cells and tumor growth was monitored for 18 days post cell injection. Error bars represent SEM (*n* = 10). Tumor area was measured and statistical analysis was performed using a mixed linear model with random intercept for animal. Difference between cell lines was highly significant (*p* < 0.0001); pairwise comparisons: *** *p* < 0.0001; * *p* = 0.0157

In order to establish an ectopic NY-BR-1 expressing tumor model in HLA-DR4tg mice we used the mammary carcinoma cell line EO771 derived from C57BL/6 mice, thus sharing a syngeneic H2 background with DR4tg mice (H2^b^). EO771 cells were transfected with a NY-BR-1 encoding expression plasmid pcDNA3.1(−)zeo-NY-BR-1 and NY-BR-1 expression among the Zeocin selected bulk culture was verified by qRT-PCR and Western blot analysis (Additional file 1: Figure S1A, B). Subsequently, single transfectant clones were raised from the bulk culture by limiting dilution resulting in a panel of EO771-derived clones showing stable NY-BR-1 protein expression (Fig. 1a). The clones EONY#9 and EONY#17 were chosen for subsequent experiments. Since formation of necrotic tumors were observed upon orthotopic application of EONY#9 and EONY#17 in initial experiments (not shown), we decided to apply these clones ectopically by s.c. injection into the flank of the mice.

**Fig. 2:**
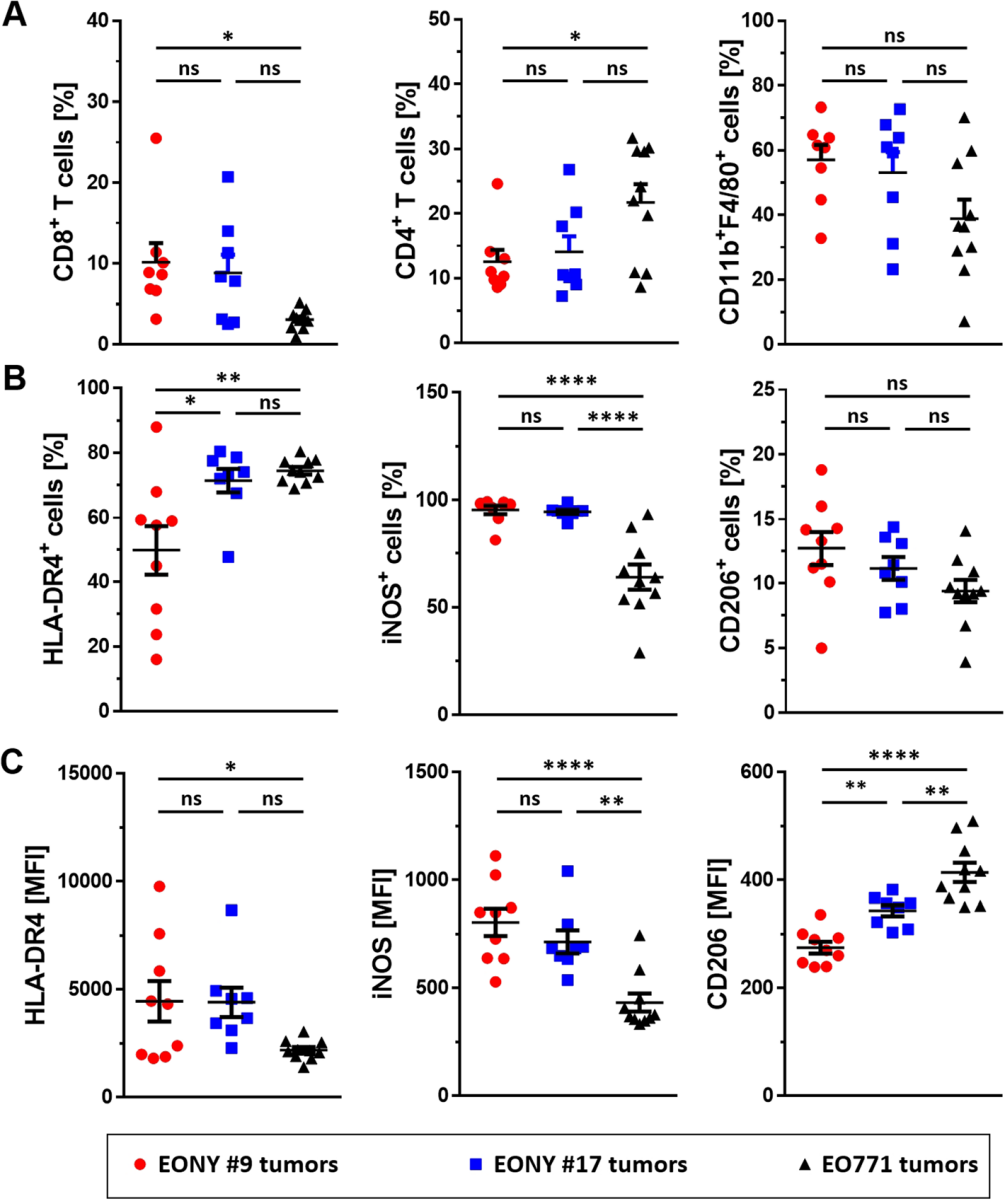
Characterization of tumor-infiltrating leukocytes in EO771 and EONY tumors. 2 × 10^5^ EO771 cells, EONY#9 cells or EONY#17 cells were injected *s.c.* into the right flank of HLA-DRB1*0401tg mice (*n* = 10). Tumor-infiltrating leukocytes were isolated 20 days post cell implantation and analyzed by flow cytometry. **a** Percentage of CD8^+^ T cells, CD4^+^ T cells and CD11b^+^F4/80^+^ macrophages among CD45^+^ leukocytes is depicted for the different tumors. **b** The frequency of TAMs expressing M1-associated markers HLA-DR4 and iNOS or M2-associated marker CD206. **c** The corresponding surface expression levels (MFI) on the positive cells is presented. Error bars depict SEM and statistical analysis performed using One-way-ANOVA with Tukey’s multiple comparisons (* *p* ≤ 0.05; ** *p* ≤ 0.01; *** *p* ≤ 0.001; **** *p* ≤ 0.0001)

When tested for their growth kinetics in DR4tg mice, EONY#9 and EONY#17 showed different growth behavior compared to parental EO771 cells (Fig. 1b). Clone EONY#9 showing strongest NY-BR-1 expression often failed to form tumors in DR4tg mice, whereas clone EONY#17 with moderate NY-BR-1 expression did still grow out, albeit at lower rate compared to parental EO771 cells. As no differences in the viability between these transfectant clones and parental EO771 cells were detected (Additional file 1: Figure S2), we concluded that NY-BR-1 expressed by the transfectant clones was immunogenic in DR4tg mice, thereby reducing outgrowth of NY-BR-1 expressing tumors. Indeed, we found significantly elevated frequencies of CD8^+^ T cells within infiltrates of tumors originating from EONY#9, compared to tumors derived from parental EO771 cells (Fig. 2a left). Consequently the relative proportion of infiltrating CD4^+^ T cells was reduced in EONY#9 tumors (Fig. 2a middle).

**Fig. 3:**
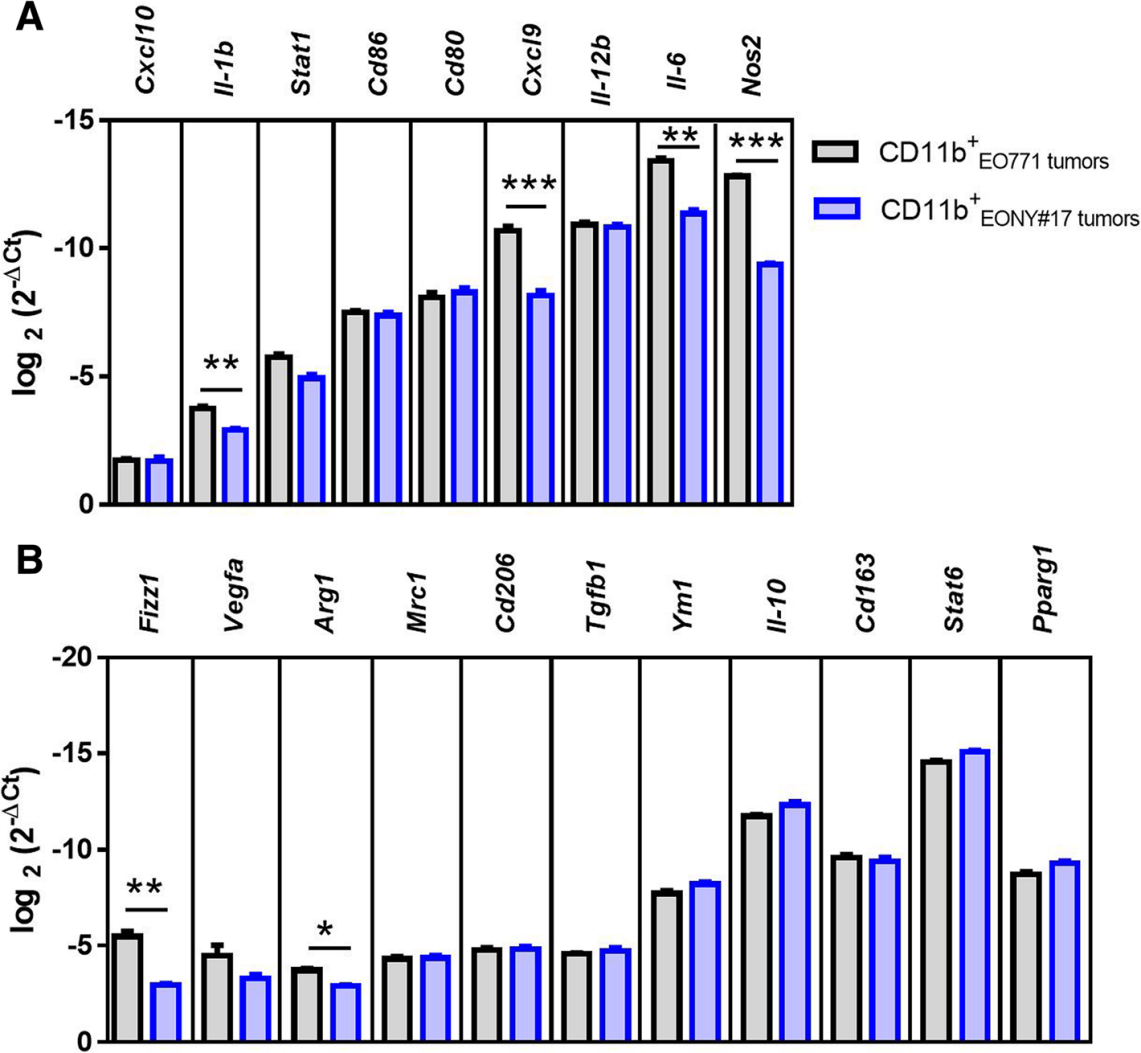
Analysis of TAM polarization in EO771 and EONY#17 tumors. Tumor infiltrating leukocytes isolated from EO771 and EONY#17 tumors were enriched for CD11b^+^ cells using anti-CD11b microbeads. The expression of various genes associated with **a** M2-like or **b** M1-like macrophages was analyzed in the isolated CD11b^+^ cells by qRT-PCR. Log fold change of each gene normalized to the house-keeping gene is shown. Error bars represent SEM and statistical analysis was performed using Student’s t test and Bonferoni-Holm adjusted *p* values were calculated (* *p* < 0.05; ** *p* < 0.01; *** *p* < 0.001)

Moreover, the proportion of CD11b^+^F4/80^+^ myeloid cells, referred to as tumor-associated macrophages (TAMs), showed a tendency of elevated frequency in NY-BR-1 expressing tumors comprising up to 70% of CD45^+^ leukocytes (Fig. 2a right). We further investigated the phenotype of these TAMs and found that EONY#9 tumors, derived from the high expresser transfectant clone #9 (Fig. 1a), contained less HLA-DR4-positive TAMs compared to tumors derived from clone EONY#17 or from parental EO771 cells (Fig. 2b, left). However, the extent of HLA-DR4 surface expression on TAMs derived from NY-BR-1 expressing transfectant clone EONY #9 exceeded HLA-DR4 surface expression of TAMs obtained from parental EO771 (Fig. 2c, left). Similarly, the frequency of iNOS producing TAMs with enhanced iNOS expression levels was significantly increased in TAMs from NY-BR-1 expressing tumors (Fig. 2b, c, middle). Focusing on CD206 as surface marker of M2-like TAMs [18] we observed that NY-BR-1 expressing tumors were infiltrated by CD206-positive macrophages to a slightly though not significant greater extent, but the level of CD206 surface expression was lower on these cells compared to EO771-derived TAMs (Fig. 2b, c right). Gene expression analyses performed on CD11b^+^ tumor infiltrating immune cells isolated from EONY#17 tumors showed enhanced expression of the M1-like markers *IL1β, Cxcl9, IL6 and Nos2* (Fig. 3a). However, expression of *Fizz1* and *Arg1* representing classical M2-like markers was elevated as well (Fig. 3b). Notably, the proportional size of the respective TIL subpopulations accumulating within the tumors did not correlate with tumor size, except for iNOS^+^ TAMs, whose proportion declined with increasing tumor size (data not shown).

Thus, s.c. transplantation of 2 × 10^5^ NY-BR-1 expressing EONY#17 cells was sufficient to give rise to subcutaneous tumors containing elevated frequencies of CD8^+^ T cells and of TAMs.

### Identification of a NY-BR-1-specific H2-D^b^-restricted CD8^+^ T cell epitope in HLA-DR4tg mice

**Fig. 4:**
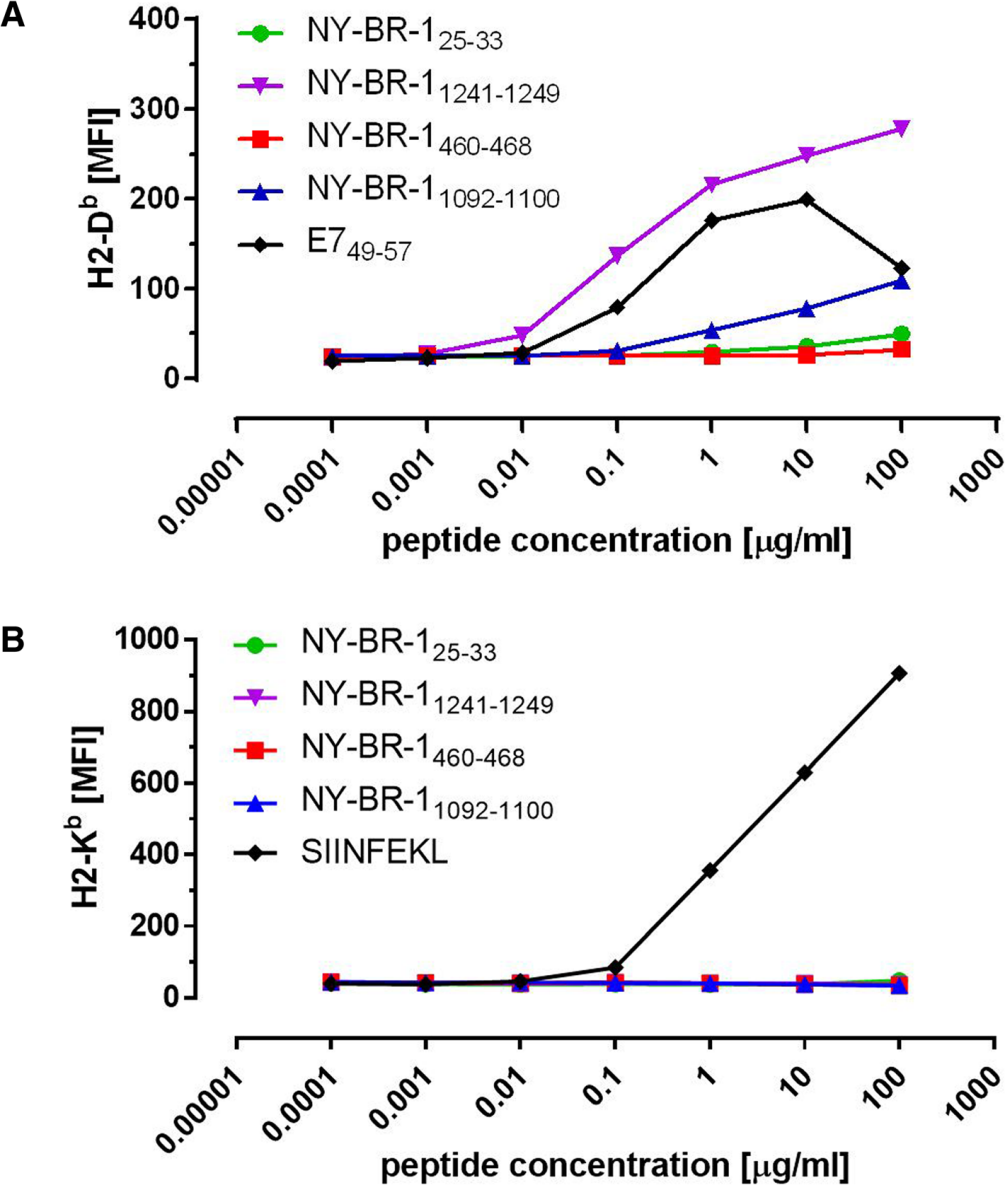
Peptide binding affinities of putative H2^b^-restricted NY-BR-1-specific CTL epitopes. Peptide binding affinity of putative CTL epitopes to H2 K^b^ and –D^b^ molecules was tested on RMA-S cells using **a** H2-D^b^-specific monoclonal antibody B22.249 or **b** H2-K^b^-specific monoclonal antibody E3–25. Surface expression of MHC I molecules stabilized by external addition of synthetic candidate epitopes was measured by flow cytometry to provide an estimate of peptide binding affinity. The H2-D^b^-restricted E7-specific epitope E7_49–57_ and the H2-K^b^-restricted OVA-specific epitope OVA_257–264_ served as positive controls

Since CD8^+^ T cells are considered as primary effector cells in tumor immune surveillance along with the fact that this T cell subset was overrepresented in NY-BR-1 expressing tumors, we set out to identify NY-BR-1-specific CD8^+^ T cell epitopes that might be involved in CTL recognition of NY-BR-1 expressing tumor cells. Upon in silico analysis applying the SYFPEITHI data base [19] on epitope containing library peptides determined previously by us [14] we selected a panel of potential H2-D^b^-restriced T cell epitopes with prediction scores greater than 23, arbitrarily set as cut off based on the scores of known CTL epitopes (Table 1). Thus, NY-BR-1_25-33_, NY-BR-1_460-468,_ NY-BR-1_1092-1100_, and NY-BR-1_1241-1249_ were generated by Fmoc chemistry and used as synthetic candidate epitopes in peptide binding assays with RMA-S cells to evaluate their binding affinity for the H2-D^b^ molecule. NY-BR-1_1241-1249_ showed strong binding affinity to the H2-D^b^ molecule, even exceeding the binding capacity observed with the E7-specific CTL epitope included as positive control. In contrast, NY-BR-1_1092-1100_ showed only marginal binding capacity, and peptides NY-BR-1_25-33_ and NY-BR-1_460-468_ completely failed to stabilize H2-D^b^ surface expression (Fig. 4a). None of the peptides tested bound to the H2-K^b^ molecule (Fig. 4b).

**Table 1:** NY-BR-1-specific H2-D^b^-restricetd CTL epitopes predicted by the SYFPEITHI database (www.syfpeithi.de)

Position	Sequence	Prediction score (D^b^)
NY-BR-1_25-33_	VYTSNDSYI	24
NY-BR-1_1241-1249_	STIYNNEVL	26
NY-BR-1_460-468_	KASANDQRF	24
NY-BR-1_1092-1100_	HTHENENYL	24

**Fig. 5:**
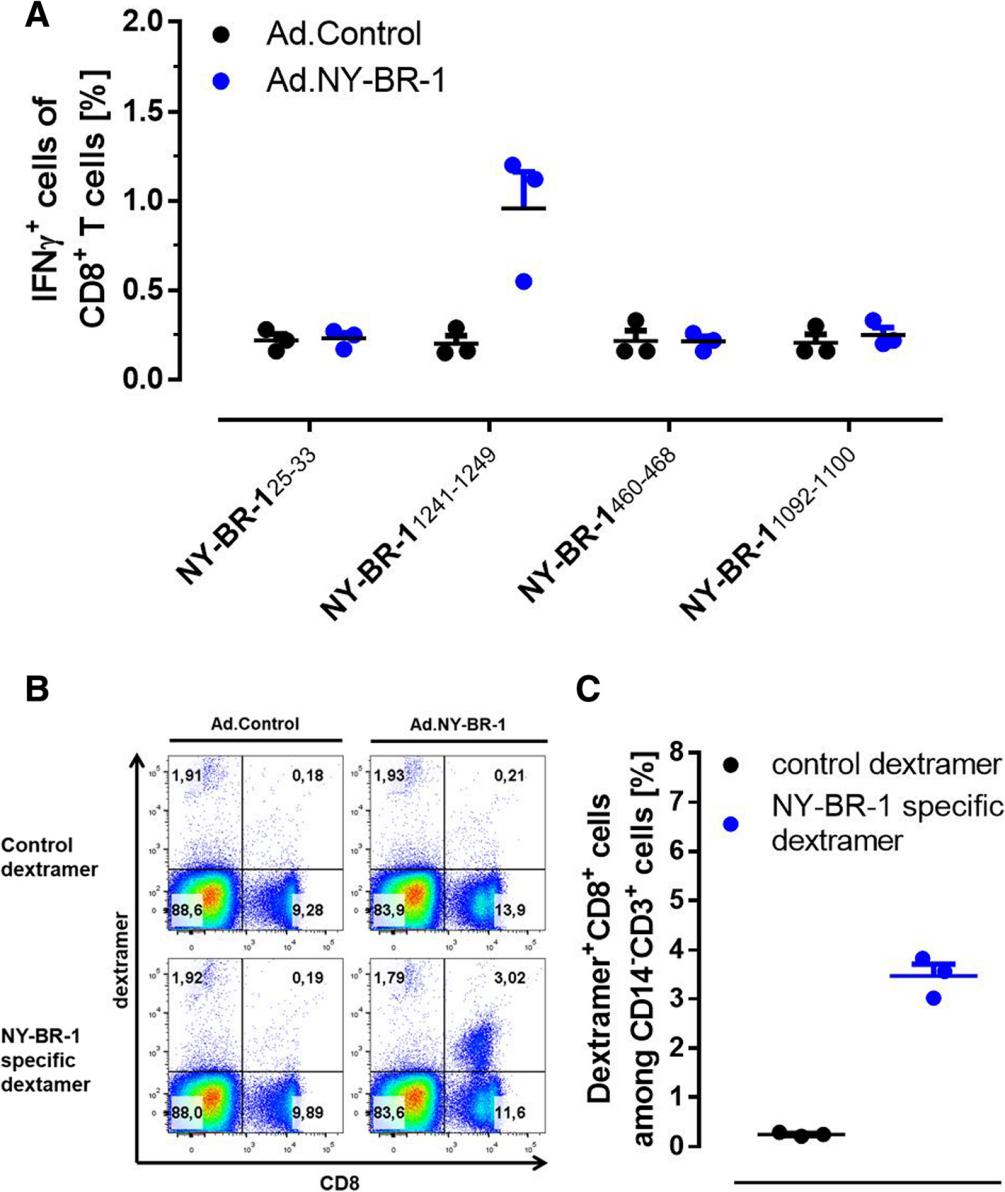
NY-BR-1_1241-1249_ is a natural H2-D^b^-restricted CTL epitope. HLA-DRB1*0401tg mice were immunized *i.p.* either with 5 × 10^8^ pfu Ad.NY-BR-1 (*n* = 3) or with 5 × 10^8^ pfu Ad. Control (*n* = 3) and splenocytes were harvested 14 days post immunization. **a** Splenocytes were incubated overnight with 5 μg/mL of synthetic peptides representing predicted NY-BR-1 epitopes. IFNγ secreting CD8^+^ T cells were analyzed by IFNγ catch assay and the percentage of CD8^+^ T cells secreting IFNγ is depicted. Immunization with Ad.NY-BR-1 resulted in CD8^+^ T cells reactive against peptides NY-BR-1_1241-1249_. **b** and **c** Splenocytes from immunized mice were stained with fluorescently labelled H2-D^b^ dextramers loaded with NY-BR-1_1241-1249_ (NY-BR-1-specific dextramer) or HPV 16 E7_49–57_ (control dextramer). **b** Proportion of dextramer^+^CD8^+^ T cells among viable CD14^−^CD3^+^ splenocytes from control mice (left panel) or from an Ad.NY-BR-1 immunized mice (right panel). **c** Representative dot plot depicting the proportion of dextramer^+^CD8^+^ T cells among viable CD14^−^CD3^+^ splenocytes from a control mouse (left panel) or from an Ad.NY-BR-1 immunized mouse (right panel)

In order to determine which of the peptides tested above would represent natural epitopes in vivo recognized by CD8^+^ T cells, HLA-DR4tg mice were immunized with Ad.NY-BR-1 followed by functional ex vivo analysis of their T cell responses against the individual candidate peptides in IFNγ catch assays 14 days later. The results revealed that NY-BR-1_1241-1249_, the peptide with the highest binding affinity to the D^b^ molecule, stimulated IFNγ responses by CD8^+^ T cells in all three mice immunized (Fig. 5a). Thus, immunization of HLA-DR4tg mice with Ad.NY-BR-1 as global NY-BR-1-specific antigen induced CD8^+^ T cell responses against NY-BR-1_1241-1249_, showing that this peptide represented a natural CTL epitope in these mice. Interestingly, the same peptide stimulated also CD4^+^ T cells, most likely due to the fact that this CTL epitope (with the exception of the first amino acid) was located within the HLA-DR4-restricted T cell epitope NY-BR-1_1242-1256_ (Additional file 1: Figure S3).

Next, we made use of a fluorochrome-conjugated H2-D^b^ dextramer displaying the newly identified CTL epitope enabling us to detect NY-BR-1_1241-1249_-specific CD8^+^ T cells directly ex vivo. Using this multimer, we found 0.3% ± 0.1% CD8^+^ dextramer^+^ spleen cells in HLA-DR4tg mice immunized with Ad.NY-BR1, as compared to 0.036% ± 0.005% in spleens of control mice. In contrast, incubation with a control dextramer displaying an irrelevant epitope did not stain spleen cells at all, thus proving specificity of our dextramer (not shown). When gating on CD3^+^ CD14^−^ splenocytes, we detected 3.0 to 3.8% CD8^+^ dextramer^+^ T cells among spleen cells of mice immunized with Ad.NY-BR-1, whereas among spleen cells from control mice or from immunized mice treated with control dextramer only 0.18 and 0.29%, of the cells appeared double positive, respectively (Fig. 5c). A representative set of dot plots showing NY-BR-1-specific CTLs among spleen cells of a control mouse and an immunized mouse is depicted in Fig. 5b.

### Immunization with Ad.NY-BR-1 mediates partial tumor protection in HLA-DR4tg mice and induces accumulation of TAMs with reduced expression of *Cxcl10*

**Fig. 6:**
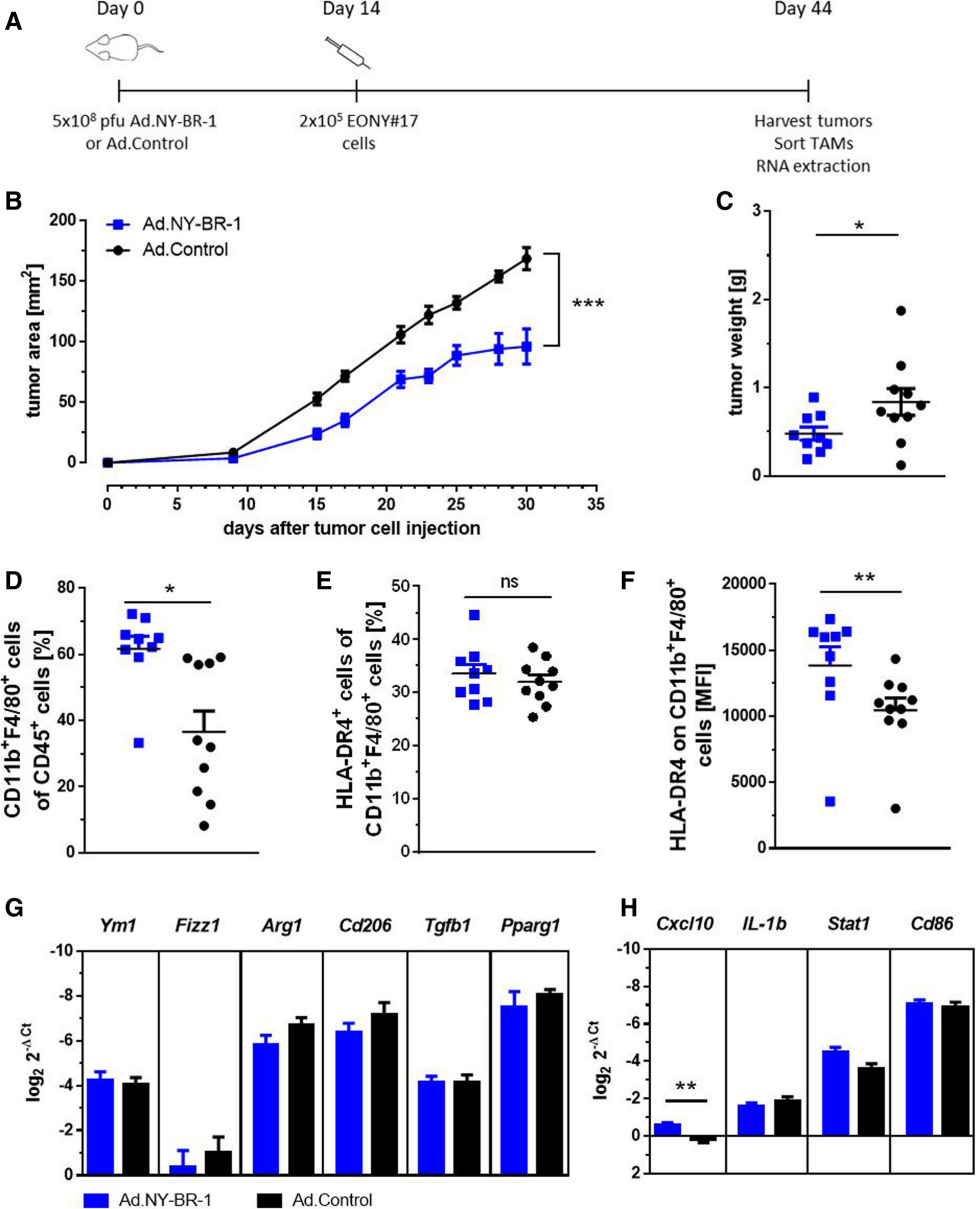
Immunization against NY-BR-1 delays tumor growth and diminishes accumulation of TAMs with reduced immunosuppressive phenotype. HLA-DRB1*0401tg mice were immunized *i.p.* with 5 × 10^8^ pfu Ad.NY-BR-1 (*n* = 10) or with 5 × 10^8^ pfu Ad. Control (*n* = 10) and 2 × 10^5^ EONY#17 cells were injected *s.c.* into the right flank 14 days post immunization. Tumor growth was monitored for 30 days followed by excision of tumors and isolation of tumor-infiltrating leukocytes. **a** Schematic representation of the experimental procedure. **b** Tumor area was measured and statistical analysis was performed using a mixed linear model with random intercept for animal. Difference in treatment was highly significant (*p* < 0.0001). Analysis was performed using SAS Version 9.4 (SAS Institute Inc., Cary, NC, USA.). **c** Tumor weight was also measured. **d**-**h** CD45^+^CD11b^+^F4/80^+^ macrophages were isolated by FACS and RNA was extracted for gene expression analysis. **d** Frequency of CD11b^+^F4/80^+^ macrophages among CD45^+^ cells, **e** frequency of HLA-DR4^+^ cells among macrophages and **f** level of HLA-DR4 surface expression on TAMs. **c**-**f** Statistical analysis was performed using Student’s t test and *p* values are indicated (* *p* ≤ 0.05; ** *p* ≤ 0.01; *** *p* ≤ 0.001; **** *p* ≤ 0.0001). Expression of **g** M2- and **h** M1-associated genes in TAMs was quantified by qRT-PCR. Log fold expression change of a gene normalized to the house-keeping gene is shown. Error bars represent SEM and Bonferoni-Holm adjusted *p* values were calculated (* *p* < 0.05; ** *p* < 0.01; *** *p* < 0.001)

Having observed that injection of Ad.NY-BR-1 induced NY-BR-1-specific T cell responses within the CD4^+^ and the CD8^+^ T cell compartments, we tested if this immunization approach could mediate protection against outgrowth of NY-BR-1 expressing tumors. Thus, HLA-DR4tg mice were immunized with Ad5.NY-BR-1 or with empty virus as control. Fourteen days later, EONY#17 cells were injected subcutaneously and tumor development was monitored for 30 days (Fig. 6a). Mice immunized with Ad5.NY-BR-1 showed decelerated tumor growth as compared to the control group (Fig. 6b). Likewise, the average tumor size and weight were significantly reduced in the group of Ad.NY-BR-1-injected mice as compared to the control values (Fig. 6c). Notably, the comparatively smaller EONY#17 tumors of Ad.NY-BR-1 immunized mice contained a higher proportion of TAMs than the tumors derived from unprotected mice (Fig. 6d). Although the proportion of HLA-DR4 expressing TAMs was independent from the vaccine applied (Fig. 6e), the extent of HLA-DR4 surface expression was significantly increased on TAMs selectively after NY-BR-1-specific immunization (Fig. 6f). This data points towards a M1-like TAM phenotype. However, expression of the classical M1 marker *Cxcl10* was down-regulated (Fig. 6h) and expression of M2-associated genes appeared unaltered (Fig. 6g).

Thus, immunization with replication-deficient, NY-BR-1-encoding adenovirus had a suppressive, though not fully inhibitory effect on outgrowth of EONY#17-derived tumors and promoted accumulation of TAMs with enhanced HLA-DR4 surface expression, accompanied by decreased expression of the M1-associated marker *Cxcl10*.

## Discussion

The current paper presents the first NY-BR1 expressing tumor model based on ectopic transplantation of the C57BL/6-derived mammary adenocarcinoma cell line EO771, stably transfected with a NY-BR-1-encoding expression vector, onto H2-compatible HLA-DR4tg mice. As NY-BR-1 represents a xeno-antigen in mice, it was not unexpected that the transplanted NY-BR-1 expressing tumor cells showed immunogenic potential promoting intra-tumoral CD8^+^ T cell infiltration and delayed tumor outgrowth. In fact, various tumor models based on xeno-antigens have been described. The most common ones use chicken ovalbumin (OVA) as target antigen, for example, in tumor models of melanoma [20], lymphoma [21] or breast cancer [22]. In some of these examples, OVA expressing transfectants showed delayed tumor growth compared to the parental tumor cell line in vivo [23–25] as observed in our NY-BR-1 expressing model, although OVA expressing tumor models without effects on tumor outgrowth have been described as well [26]. In our system, clone EONY#17 showing intermediate NY-BR-1 expression gave rise to tumors in all mice transplanted, providing the basis for the performance of tumor protection experiments. Immunization with recombinant adenovirus induced NY-BR-1-specific CTL as well as CD4^+^ T cell responses and reduced outgrowth of the NY-BR-1 expressing tumor cell line EONY#17 in DR4tg mice. Optimization of the immunization protocol, for example by inclusion of adjuvants such as CpGs or antibodies against checkpoint inhibitors in combination with adoptive T cell transfer [27–29], might further improve this protective effect, providing the basis for preclinical immunotherapy studies in this NY-BR-1 expressing tumor model.

In our study we identified the first H2-D^b^-restricted, NY-BR-1-specifc CTL epitope NY-BR-1_1241-1249_, almost entirely embedded within the HLA-DR4-restricted epitope NY-BR-1_1242-1256,_ recently identified by us [14]. Thus, immunization experiments using constructs or synthetic peptides encompassing a minimal stretch of 16 amino acids should enable simultaneous analysis of NY-BR-1-specifc CD8^+^ CTL and HLA-DR4-restricted CD4^+^ T cell responses in this mouse strain. Current HLAtg mouse strains are often engineered to lack expression of endogenous MHC molecules to prevent interference between T cell responses that are restricted by the transgenic HLA-molecule and endogenous H2 molecules respectively [30, 31]. In fact, EO-NY-derived β_2_m knock-out variants have been established [17] that could be used as parental cell lines to generate stable cell transfectant clones co-expressing NY-BR-1 in combination with the transgenic HLA molecule, resulting in NY-BR-1 expressing breast cancer cell lines that might allow performance of tumor protection and regression experiments in HLA-double transgenic mice co-expressing HLA-DR4 and HLA-A2 [32].

Breast cancer is known to be highly infiltrated by macrophages generally correlating with poor prognosis [33–37]. In our model we saw similar results with CD11b^+^F4/80^+^ macrophages representing up to 70% of CD45^+^ immune cells within the tumor. This is reminiscent to TAM infiltration rates reported in the BALB/c-derived 4 T1 breast tumor model where CD11b^+^ cells constituted up to 86% of tumor infiltrating leukocytes [38]. Performing detailed gene and protein expression analyses we found that the CD11b^+^F4/80^+^ immune cell population in NY-BR-1 expressing tumors showed enhanced expression of both, certain M1 markers genes as well as M2-associated genes. In fact, macrophages showing overlapping phenotypes thus representing neither M1 nor M2, have been described in a murine breast cancer model [39], possibly explaining the heterogenic gene expression pattern observed among TAMs in our tumor model. Alternatively, M1- and M2-like TAMs might be distributed differentially within the tumor tissue, depending on local oxygen supply with M1 accumulating preferentially in normoxic tumor areas, whereas M2-like TAMs could occur mainly at hypoxic sites [40]. We cannot judge which of these explanations might actually apply to our tumor model since our analysis was performed on the entire CD11^+^F4/80^+^ myeloid population and not on isolated M1/M2 subpopulations. Tumor antigen-specific immunization with Ad.NYBR1 induced TAMs showing upregulated HLA-DR4 surface expression levels pointing towards a possible M1-like phenotype, likely due to the induction of an IFNγ-producing Th1 response. As immunization with Ad.NY-BR-1 induced NY-BR-1-specific CD4^+^ T cells in EONY#17 tumor- bearing mice and since tumor antigen-specific CD4^+^ T cells have been reported to stimulate upregulation of MHC II expression on TAMs in the tumor microenvironment [41, 42], this might explain why the TAMs in the EONY tumors of immunized mice showed preferential expression of M1-associated markers as compared to TAMs from parental tumors.

## Conclusion

In summary, we established a NY-BR-1 expressing tumor model in HLA-DR4tg mice and introduce the first NY-BR-1-specific, H2-D^b^-resctricted CD8^+^ T cell epitope, thus allowing investigation of NY-BR-1 as a target for therapeutic vaccination approaches against breast cancer in vivo.

## Additional file

Additional file 1: **Table S1.** Antibodies used for FACS analysis. **Table S2.** Primers used for qPCR. **Figure S1.** Detection of NY-BR-1 expression in EO771/NY-BR-1 transfectants. **Figure S2.** NY-BR-1 expression does not affect the viability of EO771 transfectant clones in vitro. **Figure S3.** H2-Db restricted epitope NY-BR-11241-1249 activates CD4+ T cells. (PDF 480 kb)

## Data Availability

All data generated or analyzed during the current study are available from the corresponding author on reasonable request.

## Abbreviations

Ad.NY-BR-1: Adenoviral vector coding for NY-BR-1
C57BL/6: Mouse strain
CD: Cluster of differentiation
CTL: Cytotoxic T lymphocyte
EO771: Murine breast cancer cell line
EONY#9: Clone #9 of NY-BR-1 stably transfected EO771 cells
HLA-DR4tg: HLA-DR4 transgenic
NY-BR-1: Breast cancer antigen 1
NY-BR-1_1242-1256_: NY-BR-1 epitope comprising amino acid 1242–1256
TAMs: Tumor-associated macrophages
