# Supplementary material for: A transplantable tumor model allowing investigation of NY-BR-1-specific T cell responses in HLA-DRB1*0401 transgenic mice
Source: BMC Cancer. 2019 Sep 13;19:914. doi: 10.1186/s12885-019-6102-6 (PMC6743128; doi:10.1186/s12885-019-6102-6)
Supplement: Supplementary file 1 — Table S1. Antibodies used for FACS analysis. Table S2. Primers used for qPCR. Figure S1. Detection of NY-BR-1 expression in EO771/NY-BR-1 transfectants. Figure S2. NY-BR-1 expression does not affect the viability of EO771 transfectant clones in vitro. Figure S3. H2-Db restricted epitope NY-BR-11241-1249 activates CD4+ T cells. (PDF 480 kb) [file 12885_2019_6102_MOESM1_ESM.pdf]

*Additional file 1 to the paper by* **Das et al.:**

## A transplantable tumor model allowing investigation of NY-BR-1-specific T cell responses in HLA-DRB1*0401 transgenic mice

Parts of the results presented in this paper can be found in a dissertation by Das, 2016.

## Additional file 1: Tables S1 and S2

### Table S1: Antibodies used for FACS analysis

| specificity | fluorochrome | supplier | catalogue number |
|---|---|---|---|
| CD3 | PerCP/Cy5.5 | BioLegend | #100217 |
| CD3 | Brilliant Violet 510 | BioLegend | #100233 |
| CD4 | APC | eBioscience | #17-0041-81 |
| CD4 | PerCP/Cy5.5 | BioLegend | #100540 |
| CD4 | APC/Cy7 | BioLegend | #100414 |
| CD4 | V450 | BD Biosciences | #560470 |
| CD8 | APC | BD Pharmingen | #553035 |
| CD8 | PE/Cy7 | BioLegend | #100722 |
| CD8 | FITC | eBioscience | #11-0081-81 |
| CD11b | PerCP/Cy5.5 | BioLegend | #101228 |
| CD11c | V450 | BD Biosciences | #560521 |
| CD14 | FITC | BioLegend | #123307 |
| CD45.2 | Alexa Fluor® 488 | BioLegend | #109816 |
| CD107a | Alexa Fluor® 488 | BioLegend | #121608 |
| CD206 | Brilliant Violet 605™ | BioLegend | #141721 |
| F4/80 | Alexa Fluor® 647 | BioLegend | #123122 |
| Ly-6G/Ly-6C (Gr-1) | Alexa Fluor® 700 | BioLegend | #108422 |
| pan HLA-DR | APC/Cy7 | BioLegend | #307618 |
| I-$A^b$ | PE | BD Pharmingen | #553552 |
| I-$A^b$ | purified | BD Pharmingen | #553603 |
| NK1.1 | APC | BD Pharmingen | #561117 |
| NOS2 | PE-Cyanine7 | eBioscience | #25-5920-82 |
| IFN-γ | Brilliant Violet 421™ | BioLegend | #505830 |

Respective isotype controls were purchased from BioLegend, BD Pharmingen and BD Biosciences.

**Table S2: Primers used for qPCR**

| Primer | Sequence 5' to 3' | Reference |
|---|---|---|
| Ym1_qPCR_FP1 | CACCATGGCCAAGCTCATTCTTGT | Tatano *et al.* (2014) |
| Ym1_qPCR_RP2 | TATTGGCCTGTCCTTAGCCCAACT | |
| Fizz1_qPCR_FP3 | ACTGCCTGTGCTTACTCGTTGACT | |
| Fizz1_qPCR_RP4 | AAAGCTGGGTTCTCCACCTCTTCA | |
| IL-6_qPCR_FP5 | GTCTTCTGGAGTACCATAGC | Movahedi *et al.* (2010) |
| IL-6_qPCR_RP6 | GTCAGATACCTGACAACAGG | |
| CXCL10_qPCR_FP7 | TCTGAGTCCTCGCTCAAGTG | |
| CXCL10_qPCR_RP8 | CCTTGGGAAGATGGTGGTTA | |
| CXCL9_qPCR_FP9 | TCAACAAAAGAGCTGCCAAA | |
| CXCL9_qPCR_RP10 | GCAGAGGCCAGAAGAGAGAA | |
| IL12b_qPCR_FP11 | GAAAGACCCTGACCATCACT | |
| IL12b_qPCR_RP12 | CCTTCTCTGCAGACAGAGAC | |
| NOS2_qPCR_FP15 | GCTTCTGGTCGATGTCATGAG | |
| NOS2_qPCR_RP16 | TCCACCAGGAGATGTTGAAC | |
| VEGFA_qPCR_FP17 | CAGGCTGCTGTAACGATGAA | |
| VEGFA_qPCR_RP18 | AATGCTTTCTCCGCTCTGAA | |
| ARG1_qPCR_FP19 | TCACCTGAGCTTTGATGTCG | |
| ARG1_qPCR_RP20 | TTATGGTTACCCTCCCGTTG | |
| Mrc1_qPCR_FP21 | GCAAATGGAGCCGTCTGTGC | |
| Mrc1_qPCR_RP22 | CTCGTGGATCTCCGTGACAC | |
| CD206_qPCR_FP23 | TTGGACGGATAGATGGAGGG | Zhu *et al.* (2014) |
| CD206_qPCR_RP24 | CCAGGCAGTTGAGGAGGTTC | |
| HPRT_qPCR_FP25 | AGTACAGCCCCAAAATGGTTAAG | |
| HPRT_qPCR_RP26 | CTTAGGCTTTGTATTTGGCTTTTC | |
| bACTIN_qPCR_FP27 | TGGAATCCTGTGGCATCCATGAAAC | Davis *et al.* (2013) |
| bACTIN_qPCR_RP28 | TAAAACGCAGCTCAGTAACAGTCCG | |
| IL10_fw31 | GCTCTTACTGACTGGCATGAG | Shaul *et al.* (2010) |
| IL10_rev32 | CGCAGCTCTAGGAGCATGTG | |
| CD163_fw35 | TCCACACGTCCAGAACAGTC | Tatano *et al.* (2014) |
| CD163_rev36 | CCTTGGAAACAGAGACAGGC | |
| STAT6_fw37 | CTGGGGTGGTTTCCTCTTG | Shaul *et al.* (2010) |
| STAT6_rev38 | TGCCCGGTCTCACCTAACTA | |
| IL1β_fw39 | CTGGTGTGTGACGTTCCCATTA | |
| IL1β_rev40 | CCGACAGCACGAGGCTTT | |
| STAT1_fw41 | CTGAATATTTCCCTCCTGGG | |
| STAT1_rev42 | TCCCGTACAGATGTCCATGAT | |
| CD86_fw45 | TCTCCACGGAAACAGCATCT | |
| CD86_rev46 | CTTACGGAAGCACCCATGAT | |
| CD80_fw47 | GGCAAGGCAGCAATACCTTA | |
| CD80_rev48 | CTCTTTGTGCTGCTGATTCG | |
| TGFβ1_fw49 | AAGTTGGCATGGTAGCCCTT | |
| TGFβ1_rev50 | GCCCTGGATACCAACTATTGC | |
| PPARg_qPCR_FP51 | CGAGTCTGTGGGGATAAAGC | Provided by R. Schulz, DKFZ |
| PPARg_qPCR_RP52 | CAAACCTGATGGCATTGTGA | |

## Additional file 1: Figures S1 - S3

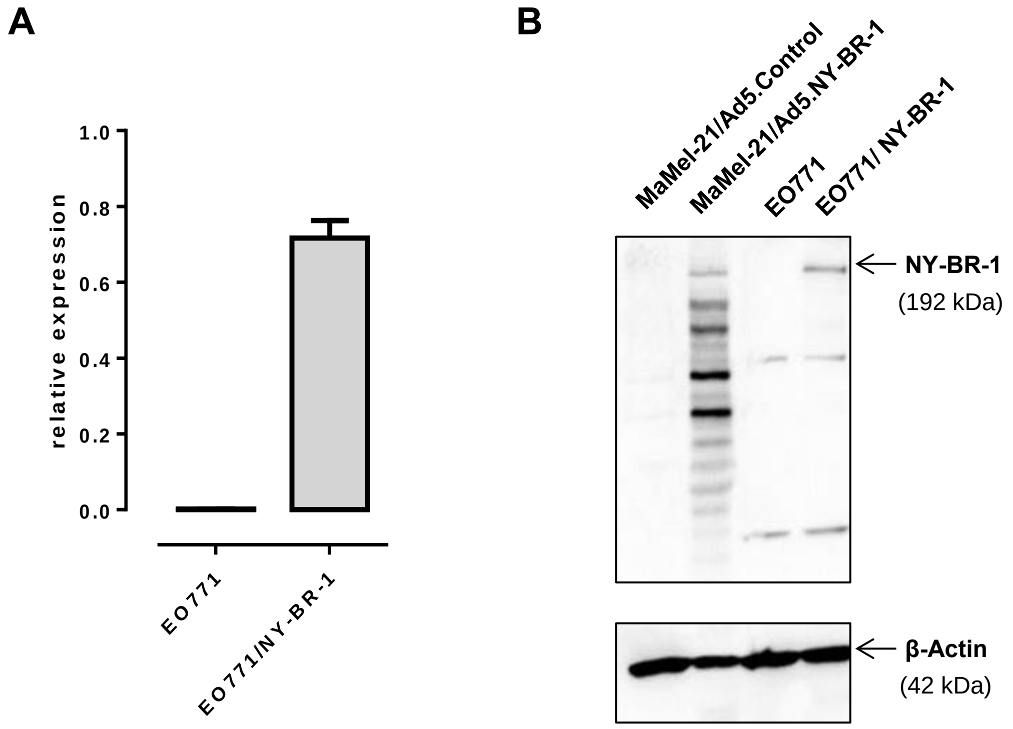


**Figure S1. Detection of NY-BR-1 expression in EO771/NY-BR-1 transfectants.** EO771 cells were transfected with a linearized plasmid pcDNA3.1-NY-BR-1 and stably transfected cells were selected with Zeocin. The expression of NY-BR-1 mRNA and protein was tested in the bulk culture prior to selection of EO771/NY-BR-1 transfectant clones. **(A)** NY-BR-1 mRNA expression was assessed by qRT-PCR relative to expression of HMBS. Error bars represent SEM (n=3). (**B**) NY-BR-1 protein was detected by Western blot thereby confirming successful transfection. The molecular mass of NY-BR-1 and β-actin is indicated on the right hand side.

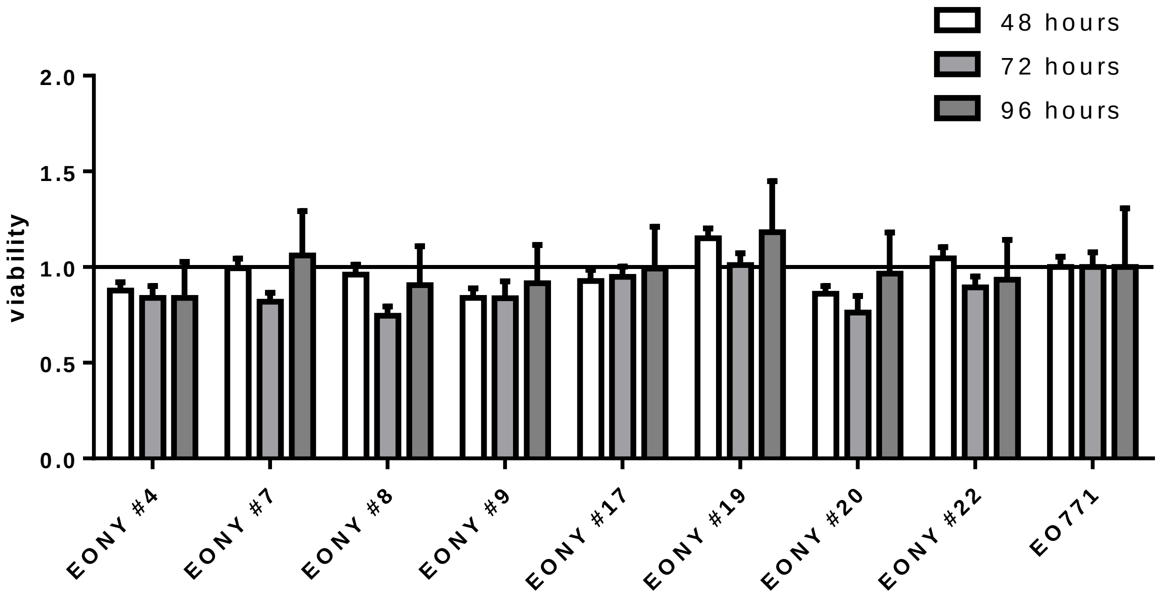


**Figure S2. NY-BR-1 expression does not affect the viability of EO771 transfectant clones *in vitro.*** Several EO771/NY-BR-1 transfectant clones obtained by limiting dilution were seeded ($2x10^4$ cells/well) and the amount of viable cells was measured after 48, 72 and 96 hours using the CellTiter-Glo® Assay. The relative luminescence measured given as relative luminescence units (RLU) is proportional to the number of viable cells. The viability of each clone normalized to parental EO771 cells is depicted for each time period.

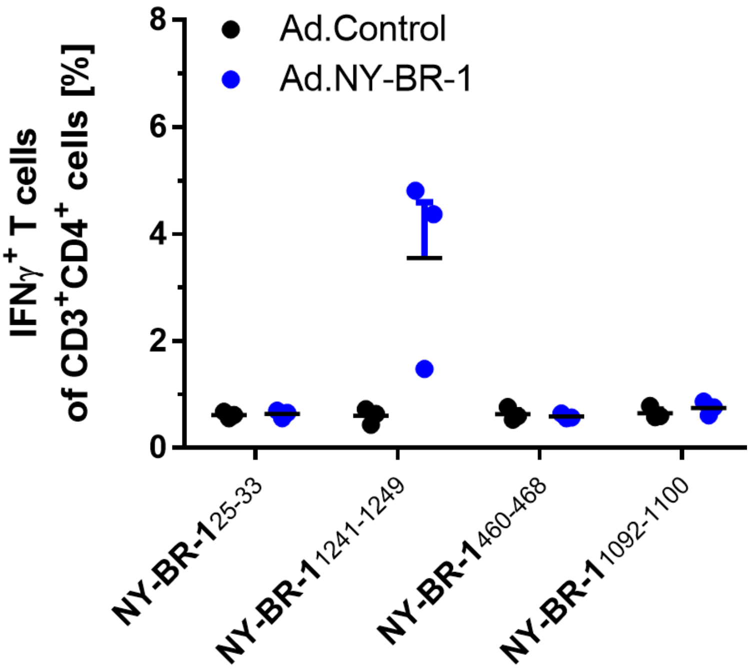


**Figure S3. H2-$D^b$ restricted epitope NY-BR-$1_{1241\text{-}1249}$ activates $CD4^+$ T cells.** HLA-DRB1*0401tg mice were immunized *i.p.* either with $5x10^8$ pfu Ad.NY-BR-1 (n=3) or with $5x10^8$ pfu Ad.Control (n=3) and splenocytes were harvested 14 days post immunization. Splenocytes were incubated overnight with 5 µg/ml synthetic peptides representing predicted NY-BR-1 epitopes and IFNγ secreting T cells were detected by IFNγ catch assay.

## References

Das K: Generation of a transplantable murine tumor model expressing the human breast cancer associated tumor antigen NY-BR-1 in HLA-DRB1*0401-transgenic mice. Dissertation, Faculty for Bioscience, Ruprecht-Karls-Universität Heidelberg, Germany, 2017.

Davis MJ, Tsang TM, Qiu Y, Dayrit JK, Freij JB, Huffnagle GB, Olszewski MA: Macrophage M1/M2 polarization dynamically adapts to changes in cytokine microenvironments in Cryptococcus neoformans infection. MBio 4:e00264-00213, 2013.

Movahedi K, Laoui D, Gysemans C, et al.: Different tumor microenvironments contain functionally distinct subsets of macrophages derived from Ly6C(high) monocytes. Cancer Res 70:5728-5739, 2010.

Shaul ME, Bennett G, Strissel KJ, Greenberg AS, Obin MS: Dynamic, M2-like remodeling phenotypes of CD11c+ adipose tissue macrophages during high-fat diet--induced obesity in mice. Diabetes 59:1171-1181, 2010.

Tatano Y, Shimizu T, Tomioka H: Unique macrophages different from M1/M2 macrophages inhibit T cell mitogenesis while upregulating Th17 polarization. Sci Rep 4:4146, 2014.

Zhu L, Yang T, Li L, et al.: TSC1 controls macrophage polarization to prevent inflammatory disease. Nature communicat*ions* 5:4696, 2014.
